# Recombinant fusion protein of Heparin-Binding Hemagglutinin Adhesin and Fibronectin Attachment Protein (rHBHA-FAP) of *Mycobacterium avium* subsp. *paratuberculosis* elicits a strong gamma interferon response in peripheral blood mononuclear cell culture

**DOI:** 10.1186/s13099-019-0317-6

**Published:** 2019-07-09

**Authors:** Vida Eraghi, Abdollah Derakhshandeh, Arsalan Hosseini, Masoud Haghkhah, Leonardo A. Sechi, Azar Motamedi Boroojeni

**Affiliations:** 1 Department of Pathobiology, School of Veterinary Medicine, Shiraz University, Shiraz, 71345-1731 Iran; 2 Sezione di Microbiologia e Virologia, Dipartimento di Scienze Biomediche, Università degli Studi di Sassari, 07100 Sassari, Italy

**Keywords:** *Mycobacterium avium* subsp. *paratuberculosis*, Johne’s disease, Chimeric protein, PBMC, IFN-γ

## Abstract

**Background:**

*Mycobacterium avium* subsp. *paratuberculosis* (MAP) is a causative agent of Johne’s disease in all ruminants worldwide. Economic problems in dairy cattle and sheep industries, public health concern, persistence of MAP in the environment and lack of effective vaccines mentioned necessity of research about various antigens to introduce as vaccine candidates. Based on MAP pathogenesis, it seems that research about the production of new recombinant proteins to stimulate cell-mediated immunity is helpful. This study describes successful expression and purification of a chimeric fusion protein which consists of Heparin-Binding Hemagglutinin Adhesin (HBHA) and high antigenic region of Fibronectin Attachment Protein (FAP-P). Triggered antigen-specific IFN-γ response of isolated PBMCs from immunized goats to rHBHA-FAP and all crude proteins of MAP (PPD), was measured by ELISA.

**Results:**

Significant increases were observed in the IFN-γ production level of peripheral blood mononuclear cells (PBMCs) stimulated by constructed chimeric protein from rHBHA-FAP and PPD vaccinated goats. Antigen-specific gamma interferon (IFN-γ) secretion in positive group (immunized by PPD) against rHBHA-FAP and test group (immunized by rHBHA-FAP) against PPD, also statistically insignificant rises between stimulation with rHBHA-FAP and PPD, suggested the potential and specificity of our chimeric protein to stimulate cell mediated immunity against MAP.

**Conclusions:**

Collectively, these results demonstrate that rHBHA-FAP elicits a strong IFN-γ production in PBMC culture. Therefore, further studies of the present product as a candidate vaccine in naturally infected animals should be conducted, to analyze its potential.

## Background

The causative agent of Johne’s disease in all ruminant species worldwide is *Mycobacterium avium* subsp. *paratuberculosis* (MAP). Chronic untreatable granulomatous enteritis in Johne’s disease leads to economic problems in dairy cattle and sheep industries that result in decreased milk, diarrhea, and weight loss [1, 2].

Although, MAP as a zoonotic pathogen has not yet been confirmed, there is some investigations about the role of MAP in public health concern. MAP can be isolated from pasteurized milk [3], children’s milk powder [4], soil and surface water [5]. Also, MAP may shed through infected animal’s feces and survive in the environment for a long period. Then the exposure of human to a contaminated environment is a potential risk [6]. There are studies about the association of MAP with Crohn’s disease [7], sarcoidosis and Blau syndrome [8], type 1 diabetes [9], Hashimoto’s thyroiditis [10], and multiple sclerosis (MS) [11]. This could explain the significant risk of MAP to public health safety. Among the approaches to reduce Johne’s disease in most countries, testing and culling practices are the most common [12], but vaccination is the best control strategy and more cost-effective [13] than other approaches. Whole-cell based vaccines, live attenuated vaccines, and inactivated vaccines were have been used until now [12] with inadequate protection. *M. avium* subsp. *paratuberculosis* binds to Microfold cells (M cells) and epithelial cells through a fibronectin bridge and mycobacterial adhesins, to cross the intestinal barrier and enter into the subepithelium. Then, MAP can be picked up by antigen presenting cells and carried to regional lymph nodes [14]. IFN-γ induced by Th_1_-mediated immune responses are play a crucial role in activating macrophages to kill intracellular MAP and protection against MAP infection [15, 16]. Gamma interferon (IFN-γ) response and antibody response can be induced by MAP. IFN-γ responses (mediated by Th_1_) detected early can lead to the controlling of MAP replication and restricted bacterial shedding; but antibody responses (mediated by Th_2_) detected late in infection which inhibit Th_1_ and are much less effective against MAP [15, 17, 18]. The best characterized mycobacterial adhesin is Heparin-binding hemagglutinin (HBHA). HBHA is located on the surface of mycobacteria and is important in the binding of mycobacteria to the epithelial cells [19] during bovine tuberculosis and Johne’s disease; it is also a major target for host humoral immune response. Some studies have demonstrated that methylated HBHA causes specific IFN-γ response in latent *M. tuberculosis* infection [20, 21]. Also, there is some reports about the induction by HBHA of both CD4^+^ and CD8^+^ T lymphocytes producing cytokines like IFN-γ in *M. tuberculosis* infection [22, 23]. The FN-binding glycoprotein family including fibronectin attachment proteins (FAPs) is important for attachment and internalization of MAP by epithelial cells and induce Th_1_ polarization and IFN-γ production in vitro [24].

Due to the global distribution of paratuberculosis and the creation of many economic problems, as well as suspicion of zoonotic nature of the MAP, using control strategies and research to identify appropriate antigens and introduce them as vaccine candidates are important. This study describes successful expression, purification, and evaluation of cellular immune response induction ability of a chimeric fusion protein which consists of HBHA and high antigenic region of FAP-P. Triggered antigen-specific IFN-γ response of isolated PBMCs from immunized goats (with our chimeric fusion protein and the crude protein fraction prepared from the culture supernatant of MAP-PPD) to rHBHA-FAP and PPD, was measured by ELISA.

## Results

### Plasmid construction and cloning

The designed chimeric gene consists of the HBHA-coding sequence, (Pro Glu)_7_ as a linker, and high antigenic region of FAP-P (amino acid 125 to 205) was synthesized and inserted in pUC57 between restriction sites of *Eco*RI and *Hin*dIII by GenScript company (USA). The transformation and propagation of the plasmid in *E. coli* DH5α was done successfully.

### Successful expression and purification of rHBHA-FAP in *E. coli* BL21 (DE3)

**Fig. 1:**
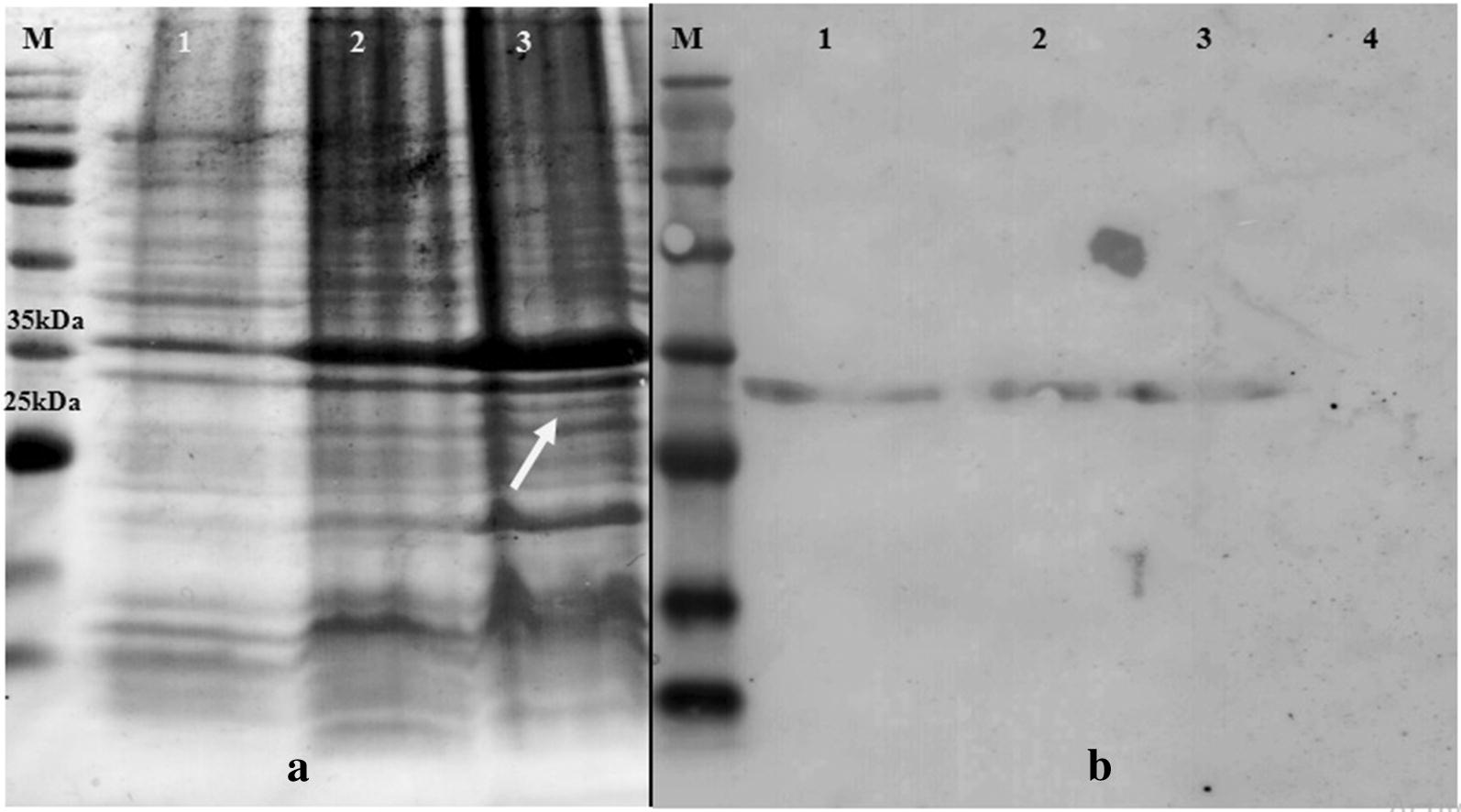
SDS-PAGE (**a**) and western blot (**b**) of chimeric rHBHA-FAP. **a** Running samples on 12% SDS-PAGE gel for choosing optimum temperature of rHBHA-FAP expression in *E. coli*. Lane M: protein ladder (CinnaGen PR911654 [SL7012]), lane 1: negative control, lane 2: expression at 30 °C, lane 3: expression at 37 °C. The white arrow indicates our desired band. **b** Western blot analysis using HRP-anti-polyhistidine. Lanes 1, 2 and 3: rHBHA-FAP with the expected size of ~ 35 kDa, lane 4: negative control

The fusion gene was successfully subcloned into the pET26b, transformed into *E. coli* BL21 (DE3), and confirmed by colony PCR. The expression of the chimeric protein is shown in Fig. 1. The desired fusion protein was determined in the medium induced by 1 mM IPTG at 37 °C after 4 h of induction and the expected size of ~ 35 kDa was obtained. Expression levels after overnight incubation or increasing IPTG did not increase significantly. Using monoclonal anti-polyhistidine-peroxidase, the desired band obtained was confirmed to be the HBHA-FAP chimeric protein by immunoblotting (Fig. 1).

Binding of polyhistidine-tagged proteins with resin was performed using Ni–NTA column (Qiagen) and desired chimeric protein was then passed over the column with elution buffer. Using imidazole in binding buffer reduced the number of proteins that bind nonspecifically to the resin; also, using Tween and 2-mercaptoethanol reduced the background signals. Purified fractions were run on a 12% SDS-PAGE gel as to check the purity of the protein extracts.

### IFN-γ response

Evaluation of cell mediated immune response to the chimeric protein was done by measuring antigen specific IFN-γ responses. The high level of secreted IFN-γ in supernatants of PBMCs in 96 h after incubation was detected. Therefore, the levels of secreted IFN-γ in 96 h after incubation were recorded in the charts. The highest level of IFN-γ was measured in supernatants of PBMCs stimulated by PHA in all groups, which indicates the ability of stimulated T-calls to secrete significant high level of IFN-γ.

**Fig. 2:**
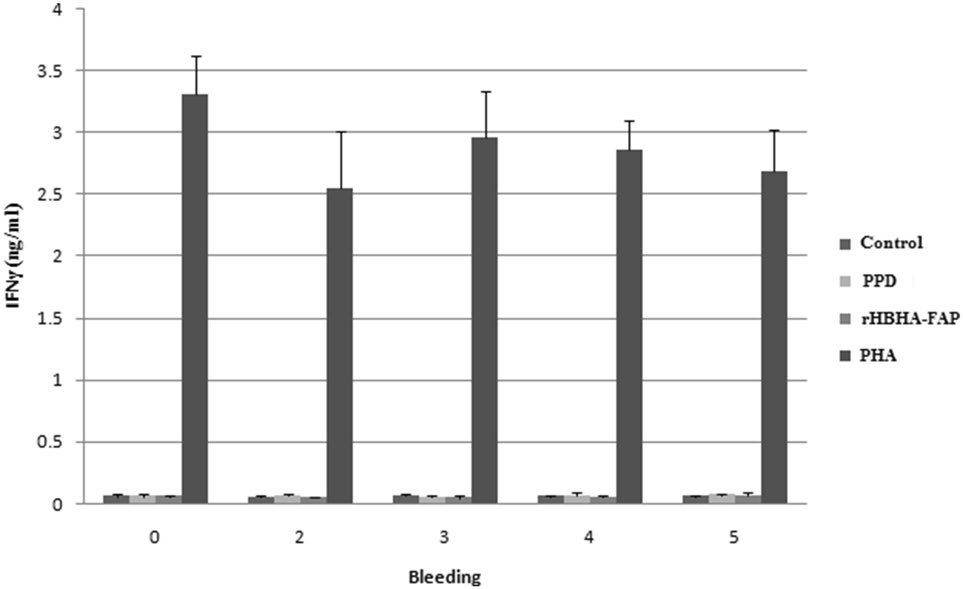
The level of IFN-γ production in supernatants of PBMCs in control negative group. PBMCs were isolated from goats immunized with adjuvant only and stimulation of isolated PBMCs was done with PHA, rHBHA-FAP and PPD 96 h after incubation. PBMCs without stimulation in all groups were considered as control. The numbers of 1 to 5, refer to first to fifth bleeding. Error bars represent standard error of the mean (SEM) between the three replicates

**Fig. 3:**
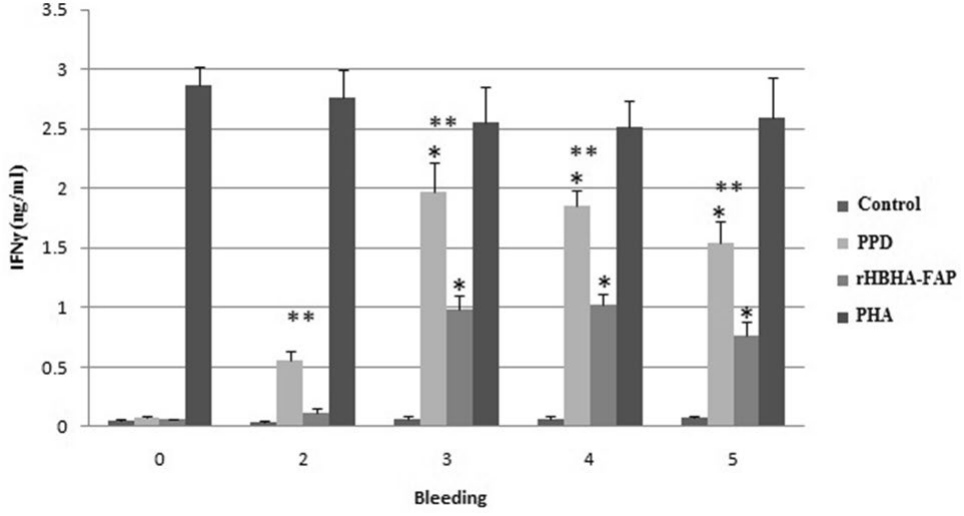
The level of IFN-γ production in supernatants of PBMCs in control positive group. PBMCs were isolated from goats immunized with PPD + adjuvant 1:1 (v/v) and stimulation of isolated PBMCs was done with PHA, rHBHA-FAP and PPD 96 h after incubation. PBMCs without stimulation in all groups were considered as control. The numbers of 1 to 5, refer to first to fifth bleeding. Error bars represent standard error of the mean (SEM) between the three replicates. *A statistically significant change as compared to the control of the same group at *p *< 0.05. **A statistically significant difference between stimulated PBMCs by PPD and rHBHA-FAP at *p *< 0.05

**Fig. 4:**
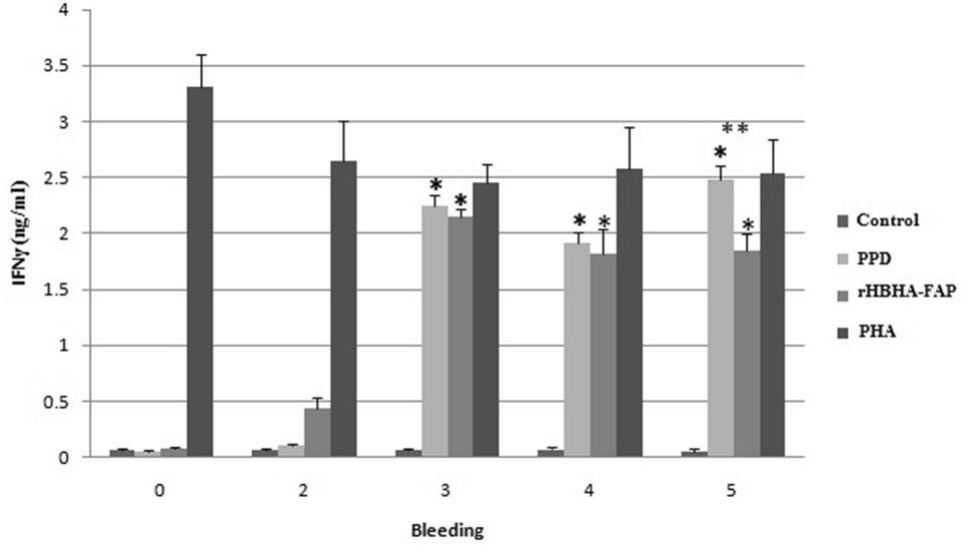
The level of IFN-γ production in supernatants of PBMCs in test group. PBMCs were isolated from goats immunized with rHBHA-FAP + adjuvant 1:1 (v/v) and stimulation of isolated PBMCs was done with PHA, rHBHA-FAP and PPD 96 h after incubation. PBMCs without stimulation in all groups were considered as control. The numbers of 1 to 5, refer to first to fifth bleeding. Error bars represent standard error of the mean (SEM) between the three replicates. *A statistically significant change as compared to the control of the same group at *p *< 0.05. **A statistically significant difference between stimulated PBMCs by PPD and rHBHA-FAP at *p *< 0.05

Although there was no significant difference in induction of IFN-γ by PPD and rHBHA-FAP within the control group (Fig. 2), levels of IFN-γ after the second booster was increased significantly in PBMCs isolated from PPD and rHBHA-FAP vaccinated goats stimulated with PPD and rHBHA-FAP (Figs. 3, 4). The response was further enhanced after the third and fourth booster in test group (Fig. 4). The highest level of secreted IFN-γ of PBMCs stimulated with PPD was observed in week three (after second booster) in positive control group (Fig. 3).

In test group, statistically significant difference between stimulated PBMCs by PPD and rHBHA-FAP was observed just in week 5 (Fig. 4); but in control positive group, the differences of IFN-γ level was statistically significant after the first vaccination (week 2, 3, 4, and 5) (Fig. 3).

## Discussion

Since MAP causes Johne’s disease with severe economic losses and zoonotical potential of the bacteria, control strategies are essential. Between control strategies, vaccination is the best [13] and attempts have been made to introduce appropriate antigens as vaccine candidates. MAP is an intracellular bacterium that can infect macrophages. It is therefore expected to stimulate cell-mediated immunity and thus cooperate in preventing disease progression [16, 25]. Then, research about the production of new recombinant proteins, evaluation of capability to IFN-γ response induction, and immunization assay are helpful. Several proteins and several antigens were introduced as vaccine candidates and some of them were tested for their potential impact [26–32]. In addition, several antigens have been reported as potent inducer of IFN-γ production, including the secreted 14-kDa protein MPP14 [33], alkyl hydroperoxidase reductase C (AhpC) and AhpD [30], the 30-kDa antigen P30 [34], a superoxide dismutase [35], the 85B antigen [36], a thiol peroxidase [37], MAP10, MAP39 and MAP41 [29] MAP1305 [38], Ag85 [39], CobT (35-kDa protein) [40], 70 kDa heat shock protein [41] and malate dehydrogenase [42]. HBHA and FAP have two crucial roles in Mycobacteria: attachment and induction of cell-mediated immunity. HBHA protein has the capability of delivering the fusion protein through surface receptors on mammalian intestinal epithelial cells and Peyer’s Patches. The full length of FAP-P has been demonstrated to have the potential to induce cell-mediated immunity in vitro [24] and in vivo [43]. Some studies have demonstrated that methylated HBHA causes specific IFN-γ response in latent *M. tuberculosis* infection [20, 21] and the induction capability of both CD4^+^ and CD8^+^ lymphocytes in *M. tuberculosis* [22]. Then, it was predicted that our chimeric protein can induce cell-mediated immunity.

In the present study, successful expression and purification of the chimeric protein consisting of HBHA and high antigenic region of FAP-P, and evaluation of its IFN-γ response induction capability in PBMCs isolated from goat models, were primarily performed. To determine whether the goat immunized with the rHBHA-FAP could induce cell mediated immunity and respond to MAP, antigen specific IFN-γ responses were measured by ELISA. The highest level of IFN-γ was measured in supernatants of PBMCs stimulated by PHA, which indicates the ability of stimulated T-cells in secreting significant high level of IFN-γ.

To check the specific response and level of secreted IFN-γ against rHBHA-FAP, immunization of goats with PPD was done to create memory T cell against all proteins of MAP.

Stimulated PBMCs isolated from pre-immunized goats and control negative group (immunized with adjuvant only) by chimeric protein and PPD did not secrete IFN-γ using ELISA. The IFN-γ production level of isolated PBMCs from test group and stimulated by chimeric protein was significantly higher than the control of the same group. Also, the differences between the level of IFN-γ was not significant between PBMCs stimulated by PPD and rHBHA-FAP except in week five. These results showed the high antigenicity of our chimeric protein as compared to stimulation of cells by crude protein fraction prepared from the culture supernatant of MAP (PPD).

The specificity of IFN-γ responses was confirmed by the significant rise in IFN-γ level after two booster injections of recombinant protein, the production of a high level of IFN-γ in PPD stimulated PBMCs isolated from rHBHA-FAP vaccinated group and the production of a high level of IFN-γ in rHBHA-FAP stimulated PBMCs isolated from PPD vaccinated group.

Antigen-specific gamma interferon (IFN-γ) secretion in positive group (immunized by all crude proteins of MAP-PPD) against rHBHA-FAP, and test group (immunized by rHBHA-FAP) against PPD, also statistically insignificant rises between stimulation with rHBHA-FAP and PPD, suggested the potential and specificity of our chimeric protein to stimulate cell mediated immunity against MAP.

## Conclusions

Collectively, our results showed that rHBHA-FAP protein is immunogenic in goats and it induces specific IFN-γ response. Therefore, because of the particularly high induction of cell-mediated immunity by this product, immunization studies of this product should be conducted as a vaccine in vivo and in vitro.

## Methods

### Bioinformatics analysis, plasmid construction and cloning methodology

In order to the construct fusion gene, the full length of HBHA HBHA-coding sequence (KC920678) which encodes adhesive part of chimeric protein was selected. Searching the full length of FAP-P (KF021287) for distinguishing high antigenic region was done using CLC software (main workbench 5.5). Eventually, one type of rigid linker between these two corresponding genes was determined in such a way that the 3D structure of HBHA was similar to its native form using TM-align (http://zhanglab.ccmb.med.umich.edu/TM-align). The final fusion gene was synthesized and inserted in pUC57 between restriction sites of *Eco*RI and *Hin*dIII by GenScript Company (USA).

*Escherichia coli* DH5α was grown at 37 °C in Luria–Bertani medium (Himedia, India) for the cloning procedure. Chemical transformation of pUC57HBHA-FAP into *E. coli* DH5α strain was done and recombinant transformants were selected by culturing on the LB medium supplemented with 50 μg/ml ampicillin. Subcloning of the fusion gene was done by digesting plasmid with *Eco*RI and *Hin*dIII (Roche, Germany), and ligating downstream of the T7 promoter into recipient expression vector of pET-26b(+). The ligation mixture was transformed into *E. coli* BL21 (DE3) and eventually, colony PCR and enzymatic digestion confirmed the presence and proper orientation of the target DNA insert in plasmid construct. For negative control, the parent pET26b without insert was also transformed similarly.

### Expression and purification of rHBHA-FAP

The overnight broth culture of recombinant colonies was inoculated into 200 ml of fresh LB medium (1:100) and the culture was shaken (150×*g*) until the OD_600_ value reached 0.6. Then, three IPTG concentrations ranging from 0.5 to 10 mM (0.5, 1 and 10 mM), and two temperatures (30 °C and 37 °C), were tried in order to choose the optimum situation for expression.

Culture pellets were collected at different time points ranging from 0 to 24 h (0 h, 1 h, 2 h, 4 h and 24 h).

The pellets were lysed by 50 μl 2× sample buffer (4% SDS, 20% glycerol, 10% 2-mercaptoethanol, 0.004% bromphenol blue and 0.125 M Tris HCl, pH approx. 6.8), heated at 90 °C for 10 min and analyzed by running them on 12% polyacrylamide gel electrophoresis under denaturing condition and were subsequently stained with Coomassie Brilliant Blue R-250 (Merck, Germany).

By using western blot, the fusion protein was identified based on expressed histidine tag. Hence, the SDS-PAGE separated proteins were transferred onto a nitrocellulose membrane and incubated with a 1:10,000 dilution of monoclonal anti-polyhistidine-peroxidase (Sigma, USA). The color development was done by using H_2_O_2_/DAB substrate/chromogen (Sigma, USA).

The conserved pelleted cells in − 20 °C were resuspended in 700 µl lysis buffer containing 7 M urea, 0.1 M sodium phosphate, and 0.01 M Tric HCl at a final pH of 8, and 1 mM proteinase inhibitor of Phenylmethanesulfonyl fluoride (Sigma, USA). The mixture was incubated at room temperature (RT) for 15 min and then insoluble debris was removed by centrifugation at 13,000*g* for 30 min at room temperature. The supernatant was added to pre-equilibrated Ni–NTA spin columns (Qiagen, Germany) and then centrifuged at 270*g* for 10 min. The wash procedure was done three times with 600 µl wash buffer containing 8 M urea, 0.1 M sodium phosphate, 0.01 M Tris.Cl, 20 mM imidazole, 2% Tween and 20 mM β-mercaptoethanol at pH of 6.3. The recombinant fusion protein was then eluted from the resin by adding 200 µl elution buffer (8 M urea, 0.1 M sodium phosphate, 0.01 M Tris.Cl and 100 mM imidazole at pH 4.5) and centrifuged at 890*g* for 2 min. To obtain soluble recombinant fusion protein and for removing urea, the collected fractions were dialyzed against four changes in PBS over the period of 36 h. Protein concentration was determined using the Bradford assay.

### Animals and immunization

Prior to the experiment, goats aged 12 months were obtained from a local farm. Fecal samples before the immunization experiments were negative for *M. avium* subsp. *paratuberculosis* by PCR targeting IS*900* gene. Three goats were immunized five times, subcutaneously in the neck, at 1-week intervals, using a volume of 1 ml of one of the following: 0.5 ml purified protein derivative (350 µg) (PPD-the crude protein fraction prepared from the culture supernatant of MAP) mixed 1:1 (v/v) with Quil A (2 mg/ml) for positive control, 0.5 ml distilled water mixed 1:1 (v/v) with the same adjuvant for negative control, and 200 µg of chimeric protein with the same adjuvant for test. Immunization and bleeding schedule are presented in Table 1. Immunization of goats with PPD was done to create memory T cell against all proteins of MAP to check the response and level of secreted IFN-γ against rHBHA-FAP.

**Table 1:** Schedule for vaccination and bleeding of goats

Time post-primary vaccination (weeks)	Procedure conducted
0	Vaccination, bleeding (5 ml, jugular vein)
1	Booster vaccination
2	Booster vaccination, bleeding (5 ml, jugular vein)
3	Booster vaccination, bleeding (5 ml, jugular vein)
4	Booster vaccination, bleeding (5 ml, jugular vein)
5	Booster vaccination, bleeding (5 ml, jugular vein)
6	Bleeding (5 ml, jugular vein)

After the experiment, all animals were transferred to animal house of school of veterinary medicine, Shiraz University.

### Primary cell cultures and IFN-γ assay

To obtain PBMC, based on the schedule (Table 1), blood samples were taken from all goats (three samples from each group). Samples were diluted 1:1 with Ca^2+^ and Mg^2+^ free phosphate-buffered saline (PBS) and centrifuged (at 1000×*g* and 20 °C for 25 min). Diluted leukocytes with PBS (1:1) were layered onto Lymphodex (Inno-Train, Germany), and centrifuged (at 800×*g* and 18 °C for 25 min). PBMCs were recovered from the buffer interface and erythrocytes were removed by incubating the cell suspension with RBC lysis buffer for 10 min at RT. Afterward, the cells were washed twice with PBS and resuspended in RPMI 1640 (GIBCO) supplemented with 10% fetal calf serum, 50 µM 2-mercaptoethanol, 1 mM sodium pyruvate, 100 µg/ml Streptomycin, and 50 µg/ml Gentamycin. Finally, 200 µl of cell suspension containing 450,000 cells was seeded onto 96-well flat-bottom plates.

PBMCs isolated from immunized goats of positive control (vaccinated with PPD), and negative control (vaccinated with adjuvant), and test (vaccinated with the chimeric protein) at various time points were stimulated either with chimeric protein (10 μg/ml), PPD (10 μg/ml), or a selective T cell mitogen (2% phytohaemagglutinin (PHA), GIBCO) in triplicates. For negative control in cell culture, the same condition of cultured cells without any stimulation was considered. Plates were incubated in 5% CO_2_ at 37 °C. Gamma interferon (IFN-γ) levels were measured in PBMC cell-free culture supernatant using commercially available ID Screen Ruminant Interferon Gamma Kit (IDvet Kit, France) in time points of 48, 72, and 96 h after incubation by the standard ELISA technique and according to the manufacturer’s instructions. The plates were read at 450 nm for optical densities using microplate reader. As a linear curve (log–log), standard curves for IFN-γ, ranging from 0.25 to 1 ng/ml, were constructed and cytokine concentrations of experimental samples were determined.

### Statistical analysis

All replicates for each group were combined to develop a mean response and error measurements were made using the standard error of the mean method. Comparisons between individual groups at each analysis time point were made using Mann–Whitney test and the p-value of < 0.05 was taken as statistically significant.

## Data Availability

The authors confirmed that all data are fully available without restriction and all relevant data are within the paper.
